# Glofitamab treatment of Richter transformation with isolated central nervous system involvement: a case report

**DOI:** 10.3389/fimmu.2025.1635589

**Published:** 2025-07-18

**Authors:** Kang Lu, Ping Chen, Xiaohui Zhang, Weicheng Zheng, Liying Zhang, Xuemei Wu, Bingzong Li

**Affiliations:** Department of Hematology, The Second Affiliated Hospital of Soochow University, Suzhou, China

**Keywords:** glofitamab, Richter transformation, DLBCL, isolated central nervous system involvement, case report

## Abstract

Diffuse large B-cell lymphoma (DLBCL) is a common subtype of Richter transformation (RT) in chronic lymphocytic leukaemia (CLL), accounting for 90–95% of all transformation cases. However, RT with isolated cerebral involvement is rare. Herein, we report the case of a 67-year-old man with DLBCL that transformed from CLL during obinutuzumab treatment. This patient developed RT with only central nervous system (CNS) involvement and no systemic features. After undergoing single-agent treatment with the CD20×CD3 bispecific antibody glofitamab, the patient regained consciousness and improved clinically. Although only one successful case has been presented, glofitamab may be considered a promising therapeutic option for patients with RT and isolated CNS involvement.

## Introduction

Richter transformation (RT) is a rare clinicopathological condition characterised by progressing indolent haematologic malignancies, such as chronic lymphocytic leukaemia (CLL), to aggressive lymphomas, often accompanied by marked multifocal lymphadenopathy and clinical deterioration. Diffuse large B-cell lymphoma (DLBCL) is the most common manifestation of RT in CLL, accounting for 90–95% of transformed cases (1). Other malignancies, including Hodgkin’s lymphoma and hairy cell leukaemia, have also been sporadically reported (2). RT incidence is low, occurring in 2–10% of patients with CLL, with an annual transformation rate of 0.5% (3). However, once RT develops, treatment becomes challenging. Compared with *de novo* DLBCL, CLL-transformed DLBCL exhibits increased chemoresistance and shortened survival (4).

Approximately one-third of *de novo* DLBCL originates from extra-nodal sites such as the gastrointestinal tract, skin, and central nervous system (CNS) (5). However, extra-nodal involvement in DLBCL-type RT is rare, especially isolated CNS involvement, with a low incidence of 0.03% (6). There are only few reports of RT with isolated CNS involvement in the literature. We found only 23 clearly reported cases since 1988 (**Table 1**). The prognosis of these patients is typically poor. Intracranial lesions result in a gradual decline in cognitive function, with most patients succumbing within 1 year. There is no standard treatment regimen for RT developing in the CNS. However, drugs that can cross the blood–brain barrier (BBB) should be prioritised. Therapeutic regimens for primary CNS lymphoma have been applied to most cases of RT with CNS involvement, including high-dose methotrexate (MTX)-based immunochemotherapy, followed by consolidation treatment with autologous stem cell transplantation or whole brain radiation therapy (WBRT). However, 15–25% of patients do not respond to high-dose MTX-based chemotherapy and 25–50% experience relapse after the initial response; it is noted that relapse rates are higher among older patients (7). Therefore, new treatment strategies for patients with RT involving the CNS need to be explored.

**Table 1:** Reported cases of Richter transformation with isolated central nervous system involvement.

Reference, year	Age, sex	Therapy	Treatment outcome
Lane P (18), 1988	45, M	BACOD, MTX	No remission after 3 months of treatment.
Neill B (19), 1989	64, M	WBRT (56 Gy)	CR after 2 years of treatment.
	79, M	WBRT (16 Gy)	Died 2 months after diagnosis.
Bayliss K (20), 1990	78, M	None	Died on the 22nd hospital day.
Mahe B (21), 1994	64, M	MTX, Teniposide, Carmustine, WBRT	Died 1.5 years after diagnosis.
	70, M	WBRT	Died 1 year after diagnosis.
Agard G (22), 1999	61, F	MTX, Ara-C, MP	Died 2 months after diagnosis.
Robak T (23), 2004	60, F	WBRT (20 Gy)	CR after 3 months of treatment.
Resende L (24), 2005	74, M	Chlorambucil, MTX, Cytarabine, WBRT (36 Gy)	Died 5 months after diagnosis.
Ghofrani M (25), 2007	64, M	R-CHOP, WBRT	Died 3 months after diagnosis.
Bagic A (26), 2007	58, F	WBRT, Rituximab	Treatment discontinuation due to loss to follow-up at 4 weeks.
Almhanna K (27), 2009	65, M	WBRT (30 Gy), ABVD	Died during the second treatment cycle.
Fløisand Y (28), 2011	58, M	MTX, Ara-C, MP, Procarbazine, WBRT (20 Gy)	CR after 1 years of treatment.
Stuplich M (29), 2012	56, M	WBRT (50 Gy), Topotecan	Died 8 months after diagnosis.
	71, F	GMALL, WBRT (50 Gy),	Treatment discontinuation due to loss to follow-up at 8 months.
Ishida F (30), 2013	66, F	Rituximab, WBRT (50 Gy)	Died 1 year after diagnosis.
Jain P (31), 2013	67, M	MTX, Ara-C, R, WBRT (24 Gy)	Died after completing one cycle of treatment.
Xu L (32), 2018	67, F	MTX, Ara-C, Dexamethasone	Died 10 weeks after diagnosis.
Pinto A (33), 2018	67, F	R-CHOP, MTX, WBRT	Significant clinical improvement was observed after 3 treatment cycles.
Cetintepe T (34), 2021	67, F	R-IDARAM	Died on the 18nd hospital day.
Pronello E (35), 2021	74, M	R, MTX	Died 3 weeks after diagnosis.
Nato Y (36), 2022	61, M	Ibrutinib, WBRT (40 Gy)	Died 5 months after diagnosis.
Wang W (37), 2022	52, M	IR-HD-MTX, Ibrutinib	CR after 6 cycles of treatment.

CR, complete response; R-CHOP, Rituximab, Cyclophosphamide, Doxorubicin, Vincristine, Prednisone; R-IDARAM, Rituximab, Ifosfamide, Cytarabine, Dexamethasone, Doxorubicin, Methotrexate; ABVD, Adriamycin, Bleomycin, Vinblastine, Dacarbazine; IR-HD-MTX, Ifosfamide, Rituximab, High-Dose Methotrexate; MTX, Methotrexate; Ara-C, Cytarabine; MP, Methylprednisolone.

Herein, we report a case of CLL transformation into DLBCL with isolated CNS involvement during orelabrutinib therapy and aim to present glofitamab as a potential therapeutic option for these patients.

## Case report

**Figure 1:**
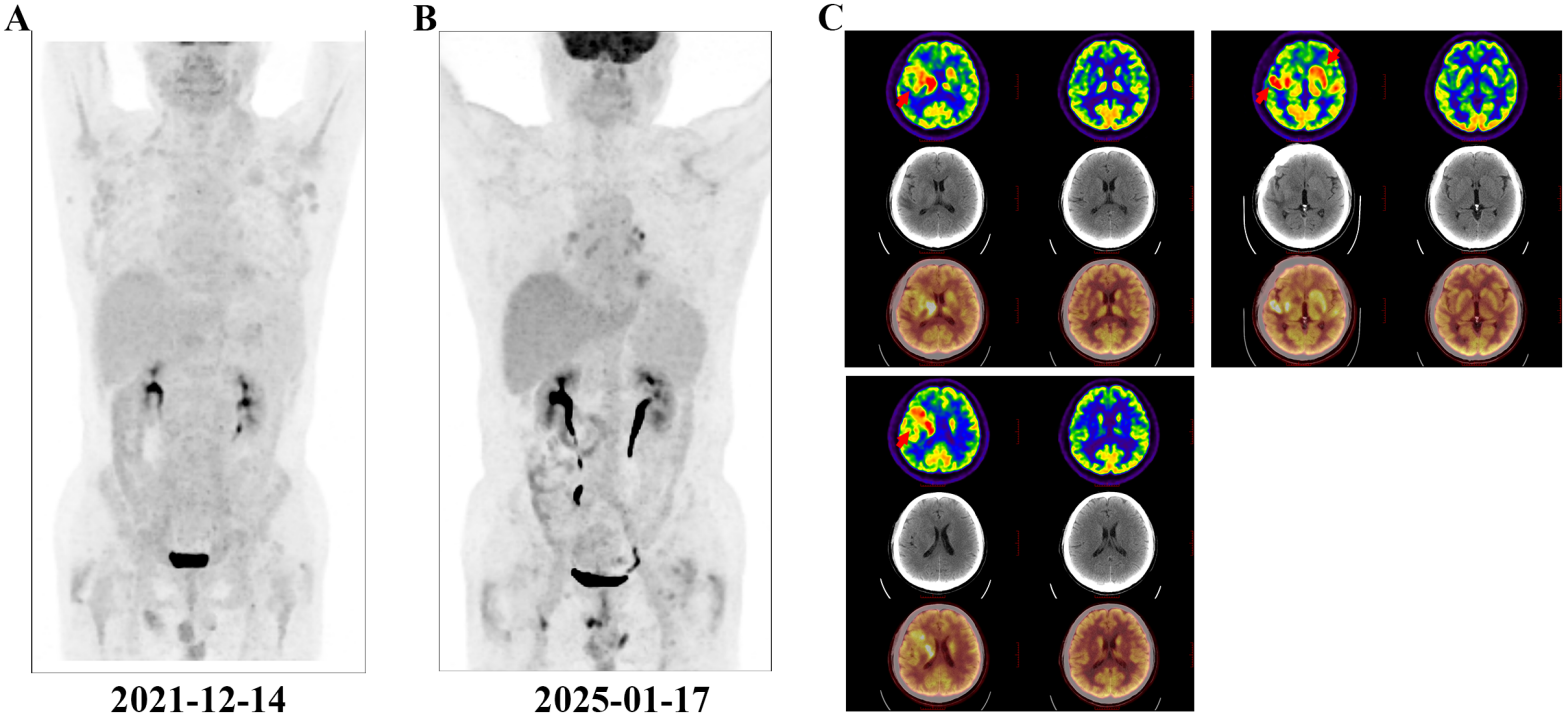
PET-CT examination. The red arrows indicate the intracranial involved lesion. **(A)** Multiple systemic foci when diagnosing CLL; **(B)** Disappearance of systemic foci when diagnosing DLBCL; **(C)** Lesions of isolated intracranial involvement when diagnosing DLBCL.

A 67-year-old man with no personal or family history of malignant tumours underwent a physical examination at a local hospital on 19 June 2019 for routine blood tests, which revealed leucocytosis (74.21×10^9/L, 95.6% lymphocytes), a haemoglobin level of 136 g/L, and platelet count of 145×10^9/L. At our clinic, imaging revealed bilateral inguinal, axillary, supraclavicular, and cervical lymphadenopathies (regions I, II, and V). Abdominal ultrasonographic findings were unremarkable. However, bone marrow morphology showed dominant proliferation of mature lymphocytes (82%). Bone marrow flow cytometry revealed CD5+, CD10- clonal B cells. Bone marrow biopsy showed active proliferation of nucleated cells in the bone marrow (approximately 80% of the haematopoietic capacity). Lymphocytes, mainly mature small lymphocytes, proliferated significantly and were distributed in a large sheet-like diffuse pattern. Immunohistochemical analysis revealed CD3 (-), CD5 (+), CD10 (-), CD20 vast (+), CD23 vast (+), CyclinD1 (-), PAX- 5 vast (+), SOX-11 (-), CD138 (-), and Ki67 (+ 5%). Chromosomes exhibited a normal karyotype. FISH revealed positive rearrangements of IgH, IgK, and IgL. Therefore, CLL (Binet system stage B, Rai system stage I) was diagnosed and classified as medium risk according to the CLL International Prognostic Index score 3. As the patient had no indications of treatment, regular follow-up visits to the haematology outpatient department were recommended. During the follow-up period, his lymphocyte counts progressively increased. By 13 December 2021, positron emission tomography-computed tomography (PET-CT) (**Figure 1A**) revealed slight to moderate abnormal increase in fluorodeoxyglucose (FDG) metabolism of multiple abnormally enlarged lymph nodes throughout the body and a slight increase in that of the bilateral humerus and femur bone marrow. Re-examination of the blood routine showed a white blood cell count of 375.5×10^9/L, lymphocyte ratio of 96.4%, haemoglobin level of 105 g/L, and platelet count of 108 g/L. Therefore, orelabrutinib 150 mg QD treatment was initiated. One year later, the patient’s blood was completely normal, and he continued orelabrutinib.

**Figure 2:**
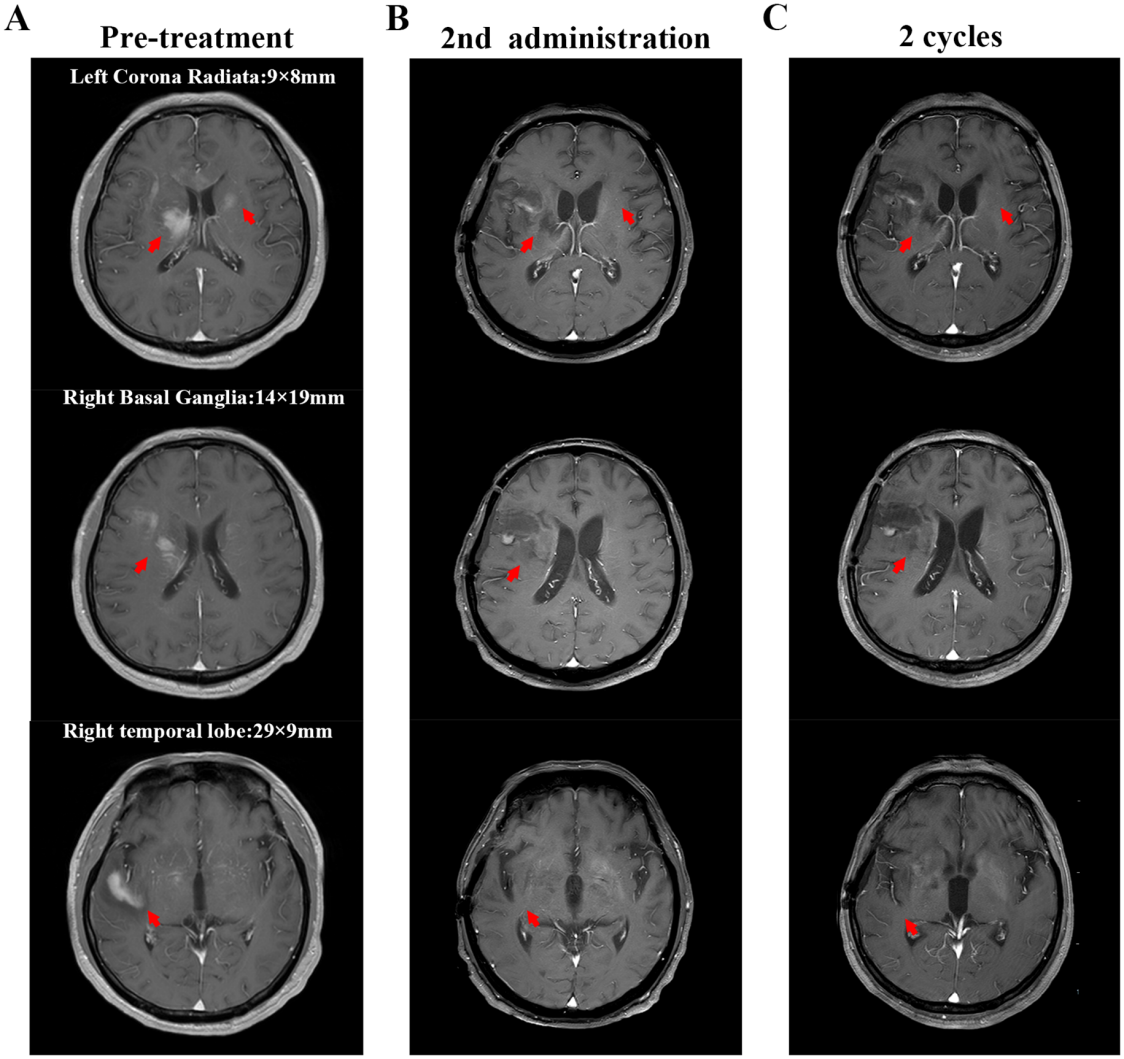
Cranial magnetic resonance imaging (MRI) findings. The red arrows indicate the intracranial lesion. **(A)** MRI scan shows the intracerebral lesions before treatment (including the right temporal lobe, right basal ganglia, and left corona radiata). The marked numbers indicate the size of the lesions; **(B)** MRI scan after the second administration of glofitamab; **(C)** MRI scan after two cycles of glofitamab administration. After treatment with glofitamab, the enhanced signal of the lesions on cranial MRI decreased, making it impossible to accurately measure their sizes.

In early 2025, the patient developed drowsiness without obvious triggers accompanied by slow movement and apathy, while independently performing daily activities. No dizziness, headache, nausea, vomiting, or limb convulsions were observed. The patient’s Glasgow Coma Scale (GCS) score was 6 points. Blood tests were unremarkable, but peripheral flow cytometry detected 6.26% CD5+CD10− clonal B cells. Cranial magnetic resonance imaging (MRI) revealed gyri-enhanced abnormal signal foci in the right basal ganglia region, bilateral corona radiata region, temporal lobe, corpus callosum knee, and compression area, suggesting intracranial involvement (**Figure 2A**). Complete PET-CT (**Figures 1B, C**) revealed slightly increased FDG metabolism in a few small retroperitoneal lymph nodes (Deauville score, 2 points) and multiple lesions throughout the body that were originally dissipated, including multiple slightly high-density nodules in the brain with abnormally elevated FDG metabolism. The larger tumour was located in the right basal ganglia, and the SUVmax on the higher part was 22.04. Chemical analysis of the cerebrospinal fluid (CSF) revealed elevated protein levels (1051 mg/L) and normal glucose levels (3.42 mmol/L); cytological analysis of CSF showed a normal total cell count (3×10^6/L), with mostly mature lymphocytes. The negative report on metagenomic next-generation sequencing of CSF ruled out an infection. Lymphoma immunotyping revealed a 70.7% lymphoid population. However, because of the small number of analysable cells, no obvious abnormalities were observed in the cell phenotype. Peripheral blood gDNA revealed pathological mutations SF3B1 p.I704F (2.4%) and DIS3 p.M163Nfs*15 (46.2%). CSF cfDNA revealed pathological mutations MYD88 p.L265P (VAF 22.2%), PRDM1 p.C19Sfs*27 (22.0%), and DIS3 p.M163Nfs*15 (48.1%). On 26 January 2025, a tumour in the right temporal lobe was punctured, and postoperative pathology showed considerable infiltration of lymphocytes. Some tumour cells were enlarged, and the proliferation index was high, suggesting tumour progression to a non-germinal centre DLBCL subtype. Immunohistochemistry results revealed tumour cells CD20 (+), GFAP (-), Ki-67 (90%+), CD79a (+), CD3 (-), CD10 (-), CD5 (-), Cyclin D1 (-), Bcl-2 (a small amount+), Bcl-6 (+), MUM1 (+), C-myc (30%), and TDT (-). Furthermore, clonal IGHV (3-11) gene rearrangements were identified in both peripheral blood and CSF, demonstrating molecular concordance with the patient’s original CLL diagnostic profile. Therefore, DLBCL of the brain was confirmed to be derived from the CLL clone.

Treatment with the R+MTX+PD-1 regimen (rituximab 700 mg d0+ methotrexate 6 g d1+ Darboshu 200 mg d4) was initiated. After two courses, the patient remained in a coma, and his general condition worsened. Thiotepa (54 mg q3w) and Selinexor (60 mg qw) were administered. The patient’s consciousness did not improve. Considering his own financial situation, the patient did not receive CAR-T therapy. Because the CD20×CD3 bispecific antibody glofitamab has shown significant efficacy and controllable safety in patients with relapsed/refractory DLBCL who are not suitable for transplantation (8, 9). In addition, individual case reports have shown that glofitamab is effective against CNS lymphoma (10, 11). After careful consideration and with the patient’s informed consent, he was treated with glofitamab, according to the standard stepwise dose escalation protocol. Since the absolute value of B lymphocytes in peripheral blood was only 15 cells/μL, obinutuzumab pretreatment was not administered.

**Figure 3:**
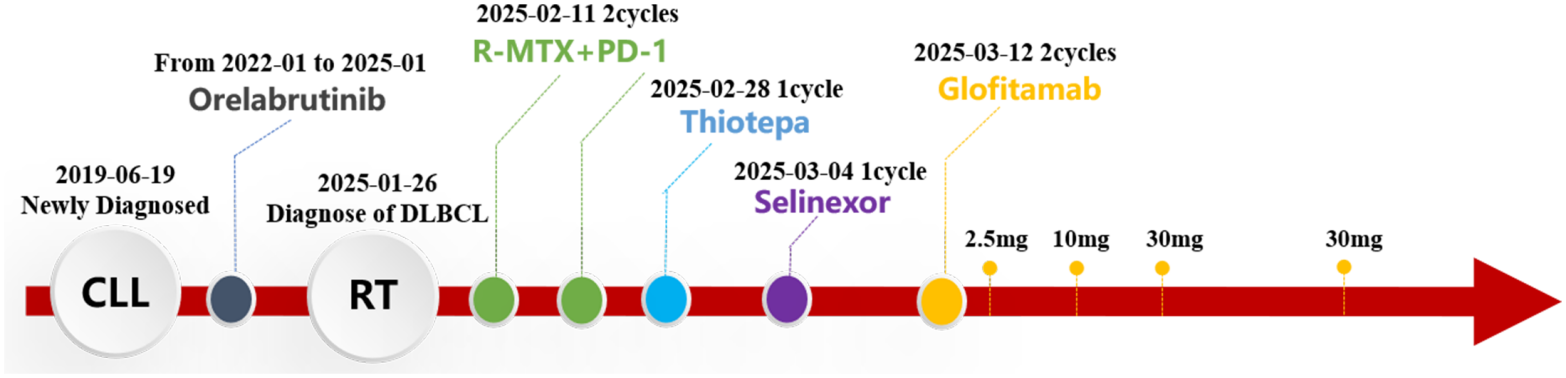
The timeline of treatment process in this case.

The patient developed grade 1 cytokine release syndrome (CRS) after the first dose of glofitamab, which mainly manifested as a persistent high fever that subsided after symptomatic treatment. No significant adverse events or unexpected complications were observed. After the second administration of glofitamab, the patient’s consciousness improved, the duration of wakefulness was prolonged, and he could engage in simple conversations. The GCS score increased to 15 points. Moreover, lymphoma immune analysis of peripheral blood showed no clonal B cells, suggesting disease remission. Re-examination of the cranial MRI (**Figure 2B**) showed that the gyrus enhancements in the brain decreased. After two cycles of glofitamab, the brain lesions shrank (**Figure 2C**). During the follow-up period, no evidence of disease recurrence and progression was observed. Treatment schedule is shown in **Figure 3**.

## Discussion

Our case highlights the exceptionally rare presentation of RT for isolated CNS-DLBCL during orelabrutinib therapy. Although DLBCL is the predominant RT subtype, cases of isolated CNS involvement are rare, and the prognosis of patients with DLBCL-type RT is typically poor, with a median overall survival of 10 months (6).

Standard DLBCL therapies yield long-term remission in 55–60% of *de novo* cases (12). However, CLL-transformed DLBCL lacks shared molecular features, suggesting its distinction (13). Moreover, the BBB limits the efficacy of systemic chemotherapy in CNS lymphomas, complicating immunotherapy.

High-BBB-penetrant agents remain the mainstay of induction. Our patient failed the R+MTX+PD-1 and salvage regimens (thiotepa/selinexor), but responded to glofitamab, which is approved for relapsed/refractory DLBCL (14). By bridging CD20+ B cells and CD3+ T cells, glofitamab forms a transient immune synapse and activates existing T cells, inducing the T cell-mediated cytotoxicity of malignant B cells in DLBCL. A multicentre Phase I/II trial (NP30179) confirmed that after pre-treatment with obinutuzumab, administering glofitamab as a single agent induced a high response rate in patients with relapsed/refractory DLBCL (9). Additionally, the efficacy and safety of glofitamab combined with other drugs, such as gemcitabine and oxaliplatin, have been verified (15).

Although glofitamab is effective against systemic B-cell lymphoma, the BBB permeability of IgG macromolecules and their role in CNS lymphoma remain to be confirmed. In this case, after switching to glofitamab monotherapy, the patient regained consciousness and the imaging lesions shrank, suggesting glofitamab’s ability in partially crossing the BBB. Moreover, two newly published studies have revealed the great potential of glofitamab in the treatment of patients with primary/secondary CNS lymphoma. Godfrey, et al. (10) found that although the average concentration of glofitamab in the CSF was only 0.1–0.4% of that in the peripheral blood, this low concentration safely alleviated the symptoms of patients with secondary CNS lymphoma. Furthermore, 5% of the CSF samples, which were collected from a patient with primary CNS lymphoma on day 2 after the fifth glofitamab administration, showed an increase in the quantity of CD25 and CD69+ T cells. Moreover, co-incubation with CD20+ lymphoma cells notably enhanced their cytotoxicity (11). The low concentration but high efficacy of glofitamab in CSF is similar to that observed with rituximab, a CD20 monoclonal antibody, indicating that a relatively low BBB penetration rate can demonstrate objective single-agent activity in CNS lymphomas (16). Preclinical studies have shown that the efficacy of CD20 bispecific antibodies is significantly higher than that of rituximab (17). Theoretically, glofitamab only requires a low CSF concentration to alleviate the clinical response in CNS lymphoma. Therefore, when the patient in this case showed no response to the R-MTX-PD1 regimen, clinical manifestations improved after switching to glofitamab.

However, existing clinical trial data show that patients treated with glofitamab have inevitably experienced some adverse events, such as cytokine release syndrome, neutropenia, anaemia, and thrombocytopenia (14). The BBB permeability of glofitamab is low. Considering that even a very low concentration of glofitamab in CSF can relieve the clinical symptoms of patients with primary/secondary CNS lymphoma (10, 11), more clinical studies and experimental data are needed to optimize the administration mode of glofitamab (such as low - dose intrathecal administration via lumbar puncture) to reduce the possibility of adverse drug events.

In summary, we reported a rare case of CLL transforming into isolated CNS-involved DLBCL via RT. The patient’s clinical symptoms improved after glofitamab monotherapy and intracranial lesions decreased, indicating glofitamab’s potential in the treatment of patients with RT and isolated CNS involvement. The follow-up period of this patient was short. Therefore, further clinical data are required to assess the therapeutic efficacy and safety of glofitamab.

## Data Availability

The original contributions presented in the study are included in the article/Supplementary Material. Further inquiries can be directed to the corresponding author.
